# Characterization of the nuclear import of the human CHD4–NuRD complex

**DOI:** 10.1242/jcs.260724

**Published:** 2023-04-06

**Authors:** Helen Hoffmeister, Simon Holzinger, Marie-Sofie Dürr, Astrid Bruckmann, Susanne Schindler, Regina Gröbner-Ferreira, Reinhard Depping, Gernot Längst

**Affiliations:** ^1^ Institute for Biochemistry, Genetics and Microbiology (Biochemistry III), University of Regensburg, 93053 Regensburg, Germany; ^2^ Institute for Biochemistry, Genetics and Microbiology (Biochemistry I), University of Regensburg, 93053 Regensburg, Germany; ^3^ Institute of Physiology, AG Hypoxia, University of Lübeck, 23562 Lübeck, Germany

**Keywords:** NuRD, Chromatin remodeling, Nuclear import

## Abstract

Chromatin remodeling enzymes form large multiprotein complexes that play central roles in regulating access to the genome. Here, we characterize the nuclear import of the human CHD4 protein. We show that CHD4 enters the nucleus by means of several importin-α proteins (1, 5, 6 and 7), but independently of importin β1. Importin α1 directly interacts with a monopartite ‘KRKR’-motif in the N-terminus of CHD4 (amino acids 304–307). However, alanine mutagenesis of this motif only leads to an ∼50% reduction in nuclear localization of CHD4, implying that there are additional import mechanisms. Interestingly, we could show that CHD4 was already associated with the nucleosome remodeling deacetylase (NuRD) core subunits, such as MTA2, HDAC1 and RbAp46 (also known as RBBP7), in the cytoplasm, suggesting an assembly of the NuRD core complex before nuclear import. We propose that, in addition to the importin-α-dependent nuclear localization signal, CHD4 is dragged into the nucleus by a ‘piggyback’ mechanism using the import signals of the associated NuRD subunits.

## Teaser

**Summary:** The human CHD4 protein has a single nuclear localization signal in the N-terminus of the protein and has already assembled into the core NuRD complex in the cytoplasm.

## INTRODUCTION

In eukaryotic cells, genomic DNA is present in a highly compacted form called chromatin. The high degree of DNA packaging requires that eukaryotic cells develop various strategies to make certain areas of chromatin accessible for DNA-dependent processes. These strategies include post-translational modifications of histones, DNA modifications, chromatin-associated RNAs and others (Bannister and Kouzarides, 2011; Breiling and Lyko, 2015; Huang and Bonner, 1965; Rodríguez-Campos and Azorín, 2007). In addition, eukaryotic cells possess a class of enzymes summarized under the term chromatin remodeling enzymes. These enzymes belong to the so-called Snf2 family of the SF2 (where SF refers to superfamily) helicases, which is further subdivided into 24 subfamilies for better categorization (Flaus et al., 2006). In addition to an ATPase domain, chromatin remodeling enzymes possess various other functional domains mainly required for chromatin association, such as chromo- or bromo-domains (Clapier and Cairns, 2009). By hydrolyzing ATP, the enzymes translocate, evict or rearrange nucleosomes on double-stranded (ds)DNA (Clapier and Cairns, 2009; Hoffmeister et al., 2017). In the context of living cells, chromatin remodelers normally act in multiprotein complexes of ten proteins or more (Clapier and Cairns, 2009).

To carry out their various DNA-dependent functions, proteins such as the chromatin remodeler CHD4 must be transported into the nucleus. Usually, this only happens when the nuclear proteins have been completely translated (Hung and Link, 2011). As a rule, nuclear localization is determined by the presence of a nuclear localization sequence (NLS). NLSs are divided into monopartite and bipartite sequences, characterized by a series of basic residues such as lysine and arginine, and which can be bound by importin-α (Robbins et al., 1991; Conti et al., 1998; Oka and Yoneda, 2018). In the case of bipartite sequences, the two basic stretches are separated by a linker region (Robbins et al., 1991; Oka and Yoneda, 2018). For proteins without such NLSs, nuclear transport is also ensured by associated binding partners according to the ‘piggyback’ principle (Steidl et al., 2004; Hunter et al., 2014).

To enable nuclear import, eukaryotic cells possess a complex transport system consisting of nuclear pore complexes (NPCs; composed of different, so-called nucleoporins), nuclear transport receptors (NTRs), and the small GTPase Ran (Oka and Yoneda, 2018). Classical nuclear transport first involves the interaction of importin-α with the NLS-containing protein in the cytoplasm. (Oka and Yoneda, 2018). Then, importin β1 binds to an importin-α protein to form a ternary complex, also known as the nuclear pore-targeting complex. This complex eventually reaches the NPCs and is guided through the hydrophobic pore with the help of the activity of importin β1. Highly abundant Ran GTPases in the nucleus finally trigger the dissociation of the complex by binding to importin β1, resulting in the nucleoplasmic release of the protein from importin-α (Oka and Yoneda, 2018).

So far, little information is available on the nuclear transport processes of chromatin remodelers. Yeast (*Saccharomyces cerevisiae*) Iswi1 has been shown to possess a bipartite NLS at the C-terminus, which appears to be highly conserved among Iswi1 homologs of other yeast species (Vasicova et al., 2013). Furthermore, the remodeling enzyme BRG1 (also known as SMARCA4) still shows nuclear localization in oocytes from Kpna6 (importin α7)-null mice, but the amount of BRG1 in the nucleus appears to be reduced overall (Hügel et al., 2014). Furthermore, it is still questionable where and when functionally active remodeling complexes will form in the transport process or route.

Given that a correct intracellular localization of a protein contributes significantly to the development of its full functional and physiological spectrum, we have therefore characterized the transport of human CHD4 – in the context of its role as the remodeling ATPase subunit of the nucleosome remodeling deacetylase (NuRD) multiprotein complex (CHD4–NuRD; Hoffmeister et al., 2017) – from the cytoplasm into the nucleus by immunocytochemical and biochemical approaches.

We show that nuclear import of the human CHD4–NuRD complex tends to be regulated independently of importin β1 through association with various importin-α proteins (1, 5, 6, and 7). Beyond that, importin α1 interacts directly with a monopartite NLS of CHD4 in the region comprising amino acids (aa) 304–307. However, alanine mutagenesis of this NLS only leads to an ∼50% reduction in the nuclear localization of CHD4, suggesting the involvement of other import mechanisms. Interestingly, we show that cytoplasmic CHD4 was already associated with NuRD core subunits, suggesting the assembly of the NuRD core complex in the cytosol prior to the passage of the nuclear pores. We, therefore, propose that CHD4 enters the nucleus via the piggyback principle (by association with NuRD core subunits) and by ‘active’ recognition of its intrinsic NLS through importins.

## RESULTS

### The N-terminal region of CHD4 directs nuclear import

**Fig. 1.**
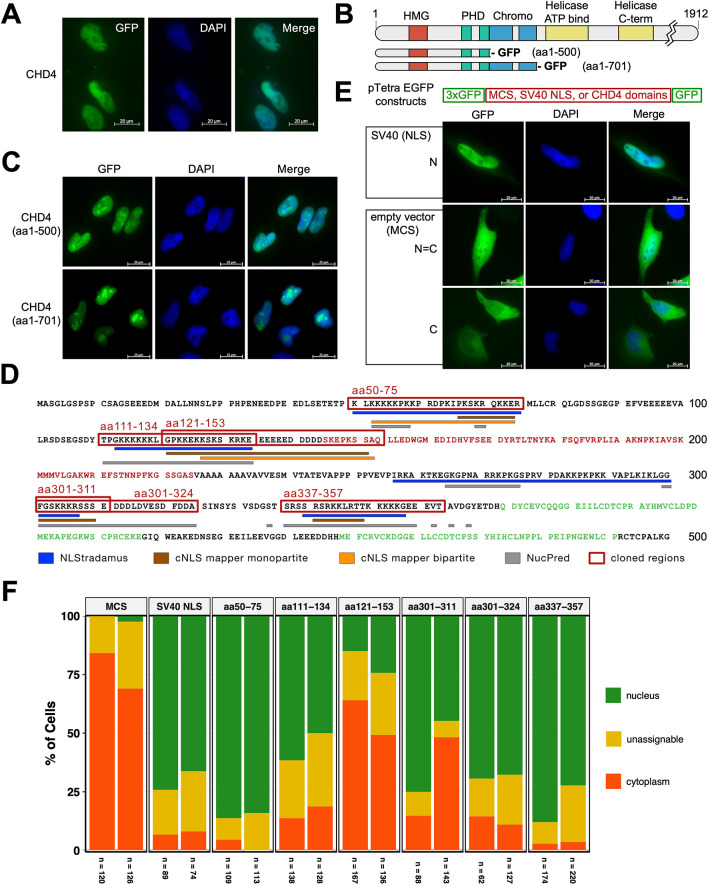
**Human CHD4 harbors an NLS motif in the N-terminal region.** (A) HeLa cells were transiently transfected with constructs encoding C-terminal GFP-tagged human wild-type CHD4. At 2 days after transfection, the cells were fixed and stained with DAPI. Scale bars: 20 µm. (B) Schematic representation of full-length CHD4 (UniProt Q14839; aa 1912) and two different C-terminally truncated versions (hCHD4 aa 1–500, and 1–701, which contains the C-terminus). (C) HeLa cells were transiently transfected with constructs encoding C-terminal GFP tagged hCHD4 aa 1–701 and aa 1–500. Two days after transfection, the cells were fixed and stained with DAPI. Scale bars: 20 µm. (D) Amino acid sequence 1–500 of human CHD4 (UniProt Q14839). Colored letters represent HMG box-like domain (red; Silva et al., 2016) and PHD domains (green, according to UniProt Q14839). Blue, orange, brown and gray lines below the sequence reflect the results of three different NLS prediction programs (NLStradamus, cNLS mapper and NucPred). Sequences used as constructs in this study are highlighted in red boxes. For more details see also Table S1. (E) Schematic representation of the pTetra-EGFP vectors used in this study. HeLa cells were transiently transfected with the empty pTetra-EGFP vector (MCS, multiple cloning site) or pTetra-EGFP vectors encoding the 4×EGFP protein with SV40 NLS or aa 50–75, 111–134, 121–153, 301–311, 301–324 or 337–357 of human CHD4 (see also Fig. S1). At 2 days after transfection, the cells were fixed and stained with DAPI. Representative examples (4×EGFP and 4×EGFP with SV40 NLS) for the classification scheme are shown (see also F). N, predominant nuclear localization; C, predominant cytoplasmic localization; N=C, no clear classification and therefore not clearly assignable to cytoplasm or nucleus (unassignable). Scale bars: 20 µm. (F) Graphical representation of the evaluation of the cells according to the classification scheme given in Fig. 1E. The diagram shows two biological replicates, with each replicate represented by a bar under which the number of cells counted/evaluated is shown. The images in A and C are representative of two biological repeats.

Immunocytochemical detection of endogenous human CHD4 or exogenous expression of GFP-tagged human (h)CHD4 revealed a prominent nuclear localization of this chromatin remodeler (Fig. 1A; Silva et al., 2016; Uhlén et al., 2005; Hoffmeister et al., 2017). The stepwise deletion of C-terminal CHD4 aa sequences and the C-terminal fusion of these deletion proteins with GFP revealed that the N-terminus up to the paired PHD domains (CHD4 aa 1–500) was sufficient to allow efficient transport of CHD4 into the nucleus of HeLa cells (Fig. 1B,C). This suggests that NLS motif(s) are located in the N-terminal region, which is consistent with previous studies, showing that the deletion of aa 1–364 leads to a visible depletion of the protein from the nucleus, resulting in a uniform distribution of the protein throughout the nucleus and the cytoplasm (Silva et al., 2016). Interestingly, the deletion of the HMG box-like domain (aa 145–225) in the context of CHD4, does not abrogate nuclear localization (Silva et al., 2016). This suggests that the nuclear localization motifs are potentially located N- and/or C-terminal to this domain. Indeed, analysis of the human CHD4 sequence (aa 1–500) by three different NLS prediction programs shows that potential NLS signaling sites are mainly located N- and also C-terminal to the HMG box-like domain (aa 1–149 and aa 217–360), whereas the domain itself has only a low NLS potential (Fig. 1D; Table S1).

To investigate whether one of the predicted signals has a nuclear localization potential, we cloned the predicted NLSs (aa 50–75; 111–134; 121–153; 301–311; 301–324; 337–357) between EGFP copy 3 and 4 of a pTetra-EGFP vector encoding a fusion protein of four modules of EGFP (4×EGFP) (Fig. 1E). The 4×EGFP protein itself localizes primarily in the cytoplasm because its size prevents it from entering the nucleus by passive diffusion (Fig. 1E; Beetz et al., 2004). It can therefore be seen as an example of a cytoplasmic-resident protein. In contrast, a 4×EGFP protein containing the SV40 NLS between EGFP copy 3 and 4 serves as a representative for a nuclear-resident protein (Fig. 1E).

All proteins were well expressed at the expected molecular mass level in HeLa cells (Fig. S1A). To determine the intracellular localization in HeLa cells, we have chosen an immunocytochemical approach by applying the following criteria according to Ding and coworkers (Ding et al., 2007). Exclusive or predominant nuclear staining (denoted as N); exclusive or predominant cytoplasmic staining (denoted as C; nuclear region rather dark, i.e. not green or significantly weaker than the cytoplasmic GFP staining), and equally strong staining in nucleus and cytoplasm, so that no clear assignment is possible (unassignable, denoted as N=C) (Fig. 1E).

**Fig. 2.**
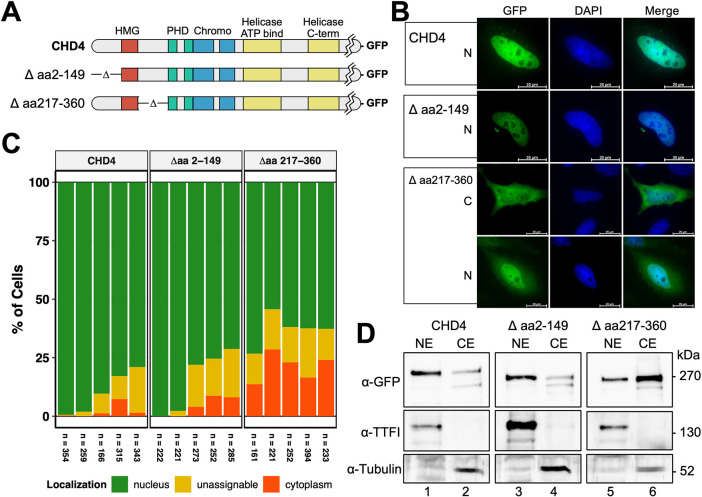
**The NLS motif in the N-terminus of hCHD4 must be located in the aa region 217–360, C-terminal of the HMG-box like domain*.*** (A) Schematic representation of the CHD4 constructs. (B) HeLa cells were transiently transfected with constructs encoding C-terminal GFP-tagged hCHD4 Δ aa 2–149, Δ aa 217–360 and hCHD4 wild-type. At 2 days after transfection, the cells were fixed and stained with DAPI. Panels show representative pictures, reflecting the preferred localization of the respective GFP fusion proteins. N, predominant nuclear localization; C, predominant cytoplasmic localization. Scale bars: 20 µm. (C) Graphical representation of the evaluation of the cells shown in B. Five biological replicates are shown, with each replicate represented by a bar under which the number of cells counted/evaluated is shown. Cells were evaluated and counted manually according to the procedure explained in Fig. 1E. (D) HeLa cells were transiently transfected with constructs encoding C-terminal GFP tagged hCHD4 Δ aa 2–149, Δ aa 217–360, and hCHD4 wild-type. At 2 days after transfection, cells were harvested and lysed for nuclear extract (NE) and cytoplasmic extract (CE) preparation. For each transfection, equal amounts of the mutually corresponding extracts were loaded according to Bradford. Western blot analysis was performed with an anti GFP antibody, whereas TTF1 and tubulin served as quality controls. Blots shown are representative of two biological repeats.

Several of the CHD4 motifs functioned as potential transport signals (Fig. 1F). The regions encompassing aa 50–75 and aa 337–357 translocate the 4×EGFP protein somewhat more efficiently to the nucleus than the classical SV40 motif and the residues 301–324 come close to the efficiency of the SV40 signal (Fig. 1E,F; Fig. S1B). The 4×EGFP proteins containing aa 111–134 and aa 301–311 were less efficient (nuclear localization ∼50%) than 4×EGFP with SV40 (Fig. 1E,F; Fig. S1B). We conclude that aa 121–153 does not drive nuclear localization, as the percentage of cells scored as having nuclear localization was similar to the percentage of cells categorized as ‘unassignable’, a value of ∼25% (Fig. 1F; Fig. 2B). Interestingly, we do not see very good agreement between the immunocytochemical data and the prediction results (Fig. 1D,F, Fig. 2B; Table S1), so the combination of both approaches does not allow us to narrow down the motif(s). This could be explained by the fact that the motifs are largely inaccessible in the structure of full-length CHD4, and that the short protein fragments might not fold properly. Moreover, it is known that both NLS and protein context contribute to specificity in importin-α binding and, consequently, nuclear import (Friedrich et al., 2006). We therefore deleted the N- and C-terminal subregions of the HMG box-like domain in the context of full length CHD4 (CHD4 Δ aa 2–149 and CHD4 Δ aa 217–360) for a rough delimitation (Fig. 2A).

The proteins were well expressed at the expected molecular mass level in HeLa cells (Fig. S2A). We observed that only the deletion of aa 217–360, C-terminal to the HMG box, led to a significant decrease in CHD4 nuclear localization to the 60% level, whereas the deletion of aa 2–149 corresponded to the values of the wild-type protein in terms of nuclear localization (Fig. 2B,C). The results were confirmed by western blot analyses of nuclear and cytoplasmic preparations of HeLa cells (Fig. 2D). Wild-type CHD4 and CHD4 Δ aa 2–149 were preferentially localized in the nucleus, whereas CHD4 Δ aa 217–360 was more enriched in the cytoplasm. TTF1 and tubulin staining ensure the purity of the respective extract preparation (Fig. 2D).

**Fig. 3.**
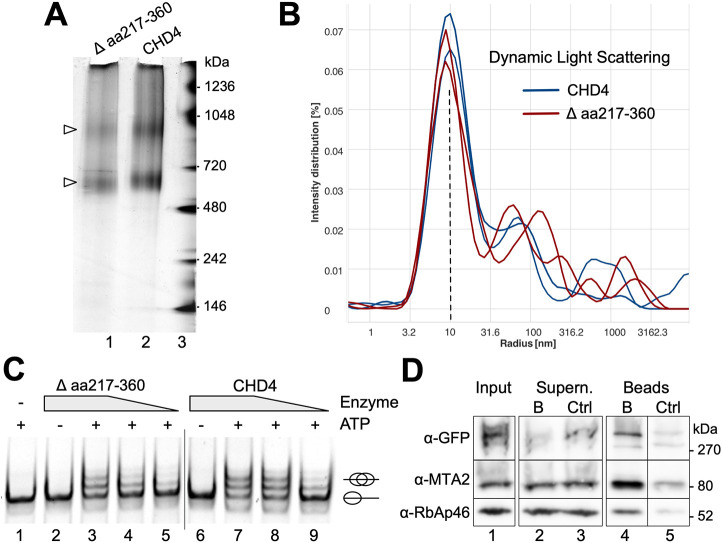
**Human CHD4 Δ aa 217–360 is still able to translocate nucleosomes and is assembled in the context of NuRD*.*** (A) A total of 1 µg of C-terminal Flag-tagged recombinant hCHD4 Δ aa 217–360 (∼ 205.2 kDa) and hCHD4 wild-type (220.5 kDa) was loaded on a 4–16% NativePAGE gel, which was subsequently stained with silver. Protein bands indicating different degrees of self-association of the respective proteins are marked with arrowheads. (B) Each 5 µM of recombinant hCHD4 Δ aa 217–360 and hCHD4 were analyzed in doublets by DLS. The hydrodynamic radius of the proteins is plotted. (C) C-terminal Flag-tagged recombinant hCHD4 Δ aa 217–360 and hCHD4 wild-type (both were added at 250 nM, 125 nM and 65 nM; enzyme) were incubated with 80 nM 0-NPS-77 nucleosomes in the presence (or absence) of 1 mM ATP for 1 h at 30°C. After stopping the reactions, all samples were loaded on a 6% native gel, which was subsequently stained with ethidium bromide. The ovals depict the position of the histone octamer on DNA. (D) HeLa cells were transiently transfected with a construct encoding C-GFP tagged hCHD4 Δ aa 217–360. At 2 days after transfection, cells were harvested and lysed for whole-cell extract (WCE) preparation. Equal amounts of WCE were subjected to immunoprecipitation experiments with GFP-Trap® A (denoted as B) and binding control agarose beads (Ctrl). Beads were finally dissolved in 2× Laemmli buffer and subjected to western blot analysis with an anti GFP antibody, anti MTA2 antibody, and RbAp46 antibody. Identical western blot analyses were performed for input and supernatant samples. The images shown are representative of two repeats.

We performed a detailed biochemical characterization to rule out that the reduced nuclear localization of CHD4 Δ aa 217–360 was a result of protein misfolding due to the deletion. The recombinant CHD4 Δ aa 217–360 exhibited the same ‘self-association’ properties as the wild-type protein, as monitored by native PAGE electrophoresis (Fig. 3A). Furthermore, we tested the homogeneity and size distribution of the protein sample by dynamic light scattering (DLS). DLS is the method of choice to determine particle sizing (Stetefeld et al., 2016). The elastic scattering of light (also known as Raleigh scattering) is used to determine the diffusion coefficients and hence the hydrodynamic radii of macromolecules. The wild-type and mutant CHD4 exhibited the same DLS profile, representing a highly monodisperse fraction with a hydrodynamic radius of ∼10 nm, corresponding to a complex with a maximum size of 1 MDa (Fig. 3B). This hydrodynamic radius correlates with the native PAGE results, showing that CHD4 and the mutant self-homomerize to form trimeric and larger complexes. Aggregates were present in very low amounts and to a similar extent for both proteins. These assays reveal that the deletion mutant resembles the wild-type CHD4, not changing its self-oligomerization and structural properties and size.

Most importantly, CHD4 Δ aa 217–360 is still functionally active, being capable of ATP-dependent nucleosome remodeling, albeit with reduced activity compared to that of the wild-type protein (Fig. 3C). The loss of activity is expected given that it has been previously shown that the entire N-terminus of CHD4 is required for efficient nucleosome remodeling (Silva et al., 2016). Furthermore, immunoprecipitation reactions from whole-cell extracts with transiently expressed C-terminal GFP tagged CHD4 Δ aa 217–360, showed that the deletion mutant still interacts with the NuRD core subunits RbAp46 (also known as RBBP7) and MTA2 (Fig. 3D), suggesting the proper folding and function of the deletion mutant. This is in agreement with another study, proposing that the C-terminal part of CHD proteins facilitates NuRD formation (Sharifi Tabar et al., 2022). In summary, we conclude that the deleted domain (aa 217–360) contains an NLS, the absence of which results in decreased nuclear import of CHD4.

### Human CHD4 harbors a NLS KRKR motif at amino acids 304–307

**Fig. 4.**
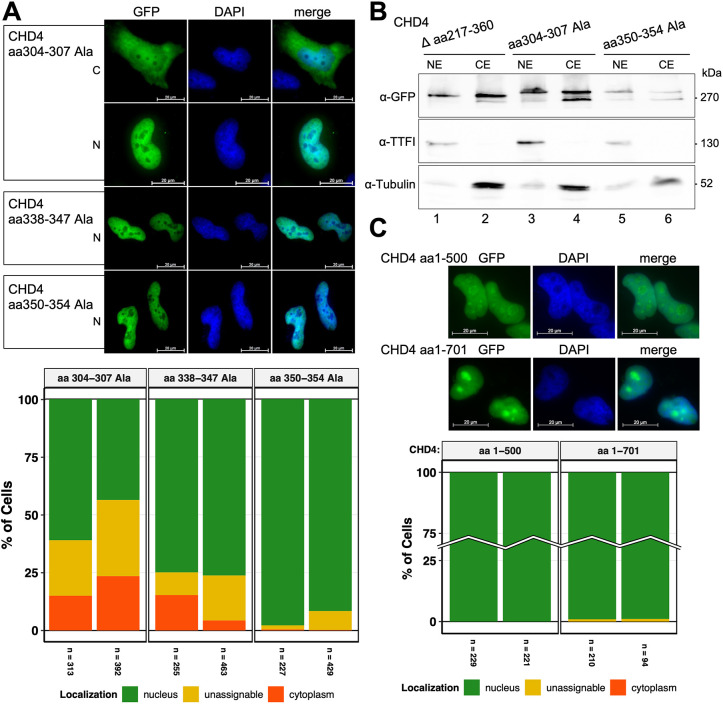
**The aa region 304–307 in hCHD4 harbors a monopartite KRKR NLS motif.** (A) HeLa cells were transiently transfected with constructs encoding C-terminal GFP tagged hCHD4 aa 304–304 Ala, aa 338–347 Ala, and aa 350–354 Ala. At 2 days after transfection, the cells were fixed and stained with DAPI. Panels show representative pictures, reflecting the preferred localization of the respective GFP fusion proteins. N, predominant nuclear localization; C, predominant cytoplasmic localization. Scale bars: 20 µm. The diagram below shows the graphical representation of the evaluation. Two biological replicates are shown, and the counted cell number is given below the bar plot. Data was analyzed as in Fig. 1E. (B) HeLa cells were transiently transfected with constructs encoding C-terminal GFP tagged hCHD4 Δ aa 217–360, hCHD4 aa 304–307 Ala, and hCHD4 aa 350–354 Ala. At 2 days after transfection, cells were harvested and lysed for nuclear extract (NE) and cytoplasmic extract (CE) preparation. For each transfection, equal amounts of the mutually corresponding extracts were loaded according to Bradford. Western blot analysis was performed with an anti-GFP antibody, whereas TTF1 and tubulin served as quality controls. Blots shown are representative of two repeats. (C) HeLa cells were transiently transfected with constructs encoding C-terminal GFP tagged hCHD4 aa 1–701 and aa 1–500 (see also Fig. 1C). At 2 days after transfection, the cells were fixed and stained with DAPI. Scale bars: 20 µm. The bar plot shows two biological replicates with the number of analyzed cells given below the plot. Data were analyzed as described in Fig. 1E.

To determine the motif responsible for the nuclear import, we have subjected lysine and arginine residues in the three predicted NLS of aa 217–360 to alanine mutagenesis (CHD4 aa 304–307 Ala), as these basic residues are characteristic for NLSs (Robbins et al., 1991; Oka and Yoneda, 2018). All three proteins were transiently expressed in HeLa cells and exhibited the expected molecular mass level (Fig. S2A). The analysis of the immunocytochemical data reveals that the motif spanning aa 304–307 (KRKR) was predominantly responsible for the transport of human CHD4 into the nucleus, whereas the motifs in aa region 338–347 and 350–354 play a minor to no role (Fig. 4A). Thus, the efficiency of nuclear import for CHD4 aa 304–307 Ala (∼ 50% nuclear localization) is comparable to that of CHD4 Δ aa 217–360 (Fig. 2C). This result was also confirmed by western blot analyses of nuclear and cytoplasmic extracts of HeLa cells, which showed that both proteins (CHD4 aa 304–307 Ala and CHD4 Δ aa 217–360) were enriched in the cytoplasmic fraction, whereas CHD4 aa 350–354 Ala accumulated in the nuclear fraction (Fig. 4B).

However, compared to the wild-type protein, the nuclear localization of both CHD4 Δ aa 217–360 and 304–307 Ala was only reduced by ∼40–50% (Figs 2C, 4A). This suggests that additional mechanisms are determining the nuclear import of CHD4. The most obvious option is the existence of additional NLSs, as has been proposed for BRCA2 (Yano et al., 2000; Hunter et al., 2014). Indeed, our NLS predictions suggest potential, additional NLSs in the region of aa 570–690 and even further C-terminal of that (Table S1). However, our experiments suggest that C-GFP CHD4 aa 1–500 is already sufficient for complete nuclear localization and proteins including additional predicted NLSs like C-GFP CHD4 aa 1–700 or the full-length protein do not show a significant difference in nuclear localization properties (Figs 1C, 4C; Fig. S2B). In agreement with reports that most of the nuclear proteins possess only a single NLS (Hunter et al., 2014), and also because ‘classical’ bipartite signals tend to follow the sequence pattern ‘KR*_X_*_10–12_KRRK’ (Ding and Sepehrimanesh, 2021; Fontes et al., 2003), we therefore thought that it was likely that hCHD4 also only possesses one monopartite NLS at aa 304–307. Even though CHD4 aa 338–347 Ala shows a slightly decreased nuclear localization (Fig. 4A), the NLS predictions do not point to a bipartite signal in the aa region 304–347 and the sequence does not quite meet the classical sequence requirements either (Fig. 1D; Table S1). Furthermore, one would expect that deletion mutants encompassing the entire bipartite signal range or mutations in the individual basic clusters would lead to equally strong effects with regard to nuclear localization deficiency, given that the clusters of a bipartite NLS are usually interdependent and indispensable (Lu et al., 2021; Vasicova et al., 2013; Zhu et al., 2015). We therefore suggest the absence of additional NLSs (besides the amino acid region aa 304–307) and propose the existence of an additional mechanism to augment nuclear import of CHD4. This would explain that mutation of the NLS (aa 304–307 Ala) still results in 40–50% of nuclear import.

### Human CHD4 is already associated with NuRD core subunits in the cytoplasm

**Fig. 5.**
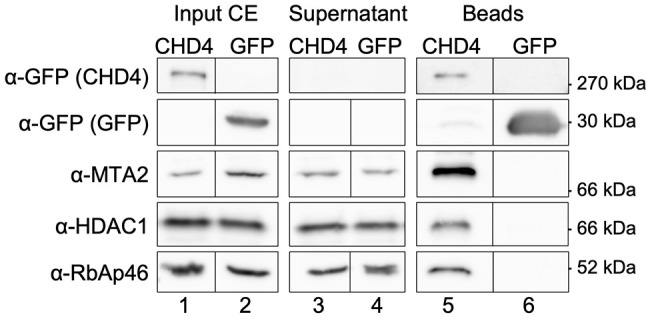
**Human CHD4 already associates with NuRD core subunits in the cytosol.** Stably transfected HEK 293 cells, expressing C-terminal GFP-tagged CHD4 wild-type and GFP upon 24 h of doxycycline induction were harvested and lysed for cytoplasmic extract (CE) preparation. Equal amounts of cytoplasmic extracts of both cell lines were subjected to immunoprecipitation with GFP-Trap® A beads. Beads were finally dissolved in 2× Laemmli buffer and subjected to western blot analysis with an anti-GFP antibody, anti-MTA2 antibody, anti-RbAp46 antibody and anti-HDAC1 antibody. Identical western blot analyses were performed for input and supernatant samples. In Fig. S3 qualitative analyses (western blot) for CE preparations can be found. Blots shown are representative of two repeats.

CHD4 is part of the NuRD complex, which comprises ten or more proteins (Clapier and Cairns, 2009; Eberharter and Becker, 2004; Hoffmeister et al., 2017). CHD4 is a nuclear protein, but our western blot analyses nevertheless reveal a small amount of CHD4 in the cytoplasm (Fig. 2D; Fig. S3), which is consistent with immunohistochemistry data from The Human Protein Atlas (https://www.proteinatlas.org/) (Pontén et al., 2008; Uhlén et al., 2005, 2010, 2015). Hence, we assessed whether the enzyme was already associated with NuRD subunits in the cytoplasm. We performed immunoprecipitation reactions from cytoplasmic extracts of stable transfected HEK 293 cells, inducible expressing wild-type CHD4 with a C-terminal GFP epitope (Fig. 5; Fig. S3). Indeed, we could show that cytoplasmic CHD4 was already associated with the NuRD core subunits HDAC1, RbAp46 and MTA2 (Fig. 5). These results show for the first time that NuRD core complexes are already assembled in the cytoplasm of mammalian cells (Fig. 5). As nuclear-resident proteins, NuRD core subunits, such as HDAC1 or MTA proteins, do possess intrinsic NLSs (Brunmeir et al., 2009; Kumar and Wang, 2016). Considering that the CHD4 Δ aa 217–360 deletion mutant associates with NuRD core subunits (Fig. 3D) and that ∼60% of HeLa cells expressing this deletion mutant show a nuclear GFP signal (Fig. 2B,C), we propose that the association of CHD4 with NuRD core subunits in the cytoplasm might act as a second, equivalent ‘driving force’ for the transport of human CHD4 into the nucleus, in addition to the KRKR motif.

### Different importin-α proteins associate with CHD4 in the context of NuRD

**Fig. 6.**
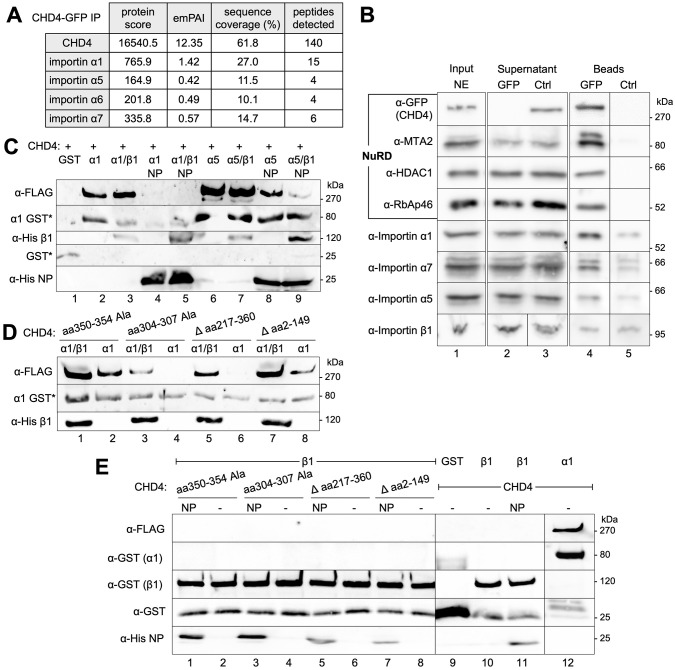
**Different importin-α proteins associate with CHD4 in the context of NuRD or with CHD4 alone*.*** (A) Stable transfected HEK 293 cells expressing C-terminal GFP tagged CHD4 wild-type upon 24 h of doxycycline induction were harvested and lysed for nuclear extract preparation. Equal amounts of nuclear extracts were subjected to immunoprecipitation with GFP-Trap® A (GFP) and binding control agarose beads (BAB; Ctrl). Beads were finally dissolved in 2× Laemmli buffer and loaded on denaturing SDS gels, which were subjected to mass spectrometric analyses. A quantitative difference in protein amount levels is reflected by the emPAI-values (exponentially modified protein abundance index) (Ishihama et al., 2005). We considered protein identification as confident if at least two unique peptides were found, and the protein received a minimum protein score of 100. Given that the control reaction (CHD4–GFP lysate on BAB beads) did not contain any detectable peptides that fulfilled the quality criteria mentioned above (see also Materials and Methods), only the values for the actual immunoprecipitation reaction [CHD4-GFP lysate on GFP-TrapA beads (GFP)] are shown. All raw values and data for both immunoprecipitation reactions can be found in Table S2. (B) Western blot analysis of immunoprecipitation reactions, performed as described in A with an anti-GFP antibody, anti-MTA2 antibody, anti RbAp46 antibody, anti HDAC1 antibody, and antibodies directed against importin α1, α5 and α7 or importin β1. Identical western blot analyses were performed for input and supernatant samples. (C) GST alone or GST-importin α1 and 5 (α1 and α5) were immobilized in the absence or presence of His-tagged importin-β (β1) or nucleoplasmin (NP) to glutathione–Sepharose beads. Subsequently, purified CHD4–C-Flag was allowed to bind to the immobilized fusion proteins. The bead fractions were resolved on SDS gels and analyzed by western blotting with an antibody directed against the Flag epitope (CHD4) or an anti-His antibody (importin-β/nucleoplasmin). Note that the anti-Flag antibody also detects (non-specifically) the precipitated GST (fusion) proteins (labeled with an asterisk). (D) GST–importin α1 (α1) was immobilized in the absence or presence of His-tagged importin-β (β1) to glutathione–Sepharose beads. Subsequently, purified CHD4-C-Flag Δ aa 2–149, CHD4-C-Flag Δ aa 217–360, CHD4-C-Flag aa 304–307 Ala, or CHD4 C-Flag aa 350–354 Ala were allowed to bind to the immobilized fusion proteins. The bead fractions were resolved on SDS gels and analyzed by western blot with an antibody directed against the Flag epitope (CHD4) and an anti-His antibody (importin-β). Also note that the anti-Flag antibody also detects (non-specifically) the precipitated GST tagged importin-α proteins (labeled with an asterisk). (E) GST alone, GST–importin α1 (α1), or GST–importin β1 were immobilized in the absence or presence of nucleoplasmin (NP) to glutathione–Sepharose beads. Subsequently, purified wild-type CHD4-C-Flag, CHD4-C-Flag Δ aa 2–149, CHD4-C-Flag Δ 217–360, CHD4-C-Flag aa 304–307 Ala, or CHD4 C-Flag aa 350–354 Ala were allowed to bind to the immobilized fusion proteins. The bead fractions were resolved on SDS gels and analyzed by western blotting with an antibody directed against the Flag epitope (CHD4 proteins), an anti-GST antibody (GST–importin α1 and GST–importin β1, or GST itself), or an anti-His antibody (nucleoplasmin). Note that there is a small amount of ‘co-purified’ GST in all GST fusion protein fractions (lanes 1–8, 10–12), which is detected by the GST antibody as well. However, the signal intensity for GST (lane 9) is greatly enhanced in comparison to the GST by product in those lanes. Control experiments in Fig. S4B show that neither hCHD4 wild-type, nor the various hCHD4 deletion or alanine mutants bind non-specifically to glutathione–Sepharose beads, saturated with immobilized GST. Blots shown are representative of two repeats.

Finally, to identify the transport proteins involved in nuclear import, we performed immunoprecipitation experiments with C-terminally GFP-tagged CHD4 from nuclear extracts of stably transfected HEK 293 cells. Then, the CHD4-associated proteins were analyzed by mass spectrometry (Fig. 6A; Table S2). As expected, we identified NuRD core subunits, such as HDAC1 and HDAC2, MTA1–MTA3 and many other known NuRD-associated proteins, as co-precipitating with CHD4 (Fig. 6B; Table S2), which is consistent with previous data published by our laboratory (Hoffmeister et al., 2017). In addition, the nuclear import factors importin α1, α5, α6 and α7 were associated with CHD4–NuRD (Fig. 6A; Table S2). The association of importin α1, α5 and α7 with CHD4 was also confirmed by western blotting (Fig. 6B). For importin β proteins, we obtained only few and poor quality (peptide) signals in the mass spectrometric analysis, and the western blot analysis also does not show a specific enrichment for the importin β1 protein (Fig. 6B; Table S2). This might be due to the fact that importin β proteins only bind the importin-α nuclear protein complex as the third component and therefore presumably remain in the transport complex for a shorter time than importin-α proteins (Oka and Yoneda, 2018).

We asked next, whether importins could also interact directly with CHD4 by performing *in vitro* binding studies according to Depping and colleagues (Depping et al., 2008). Indeed, we observed a direct interaction of recombinant CHD4 with recombinant importin α1 and α5, which could be competed out by adding nucleoplasmin (Fig. 6C; Fig. S4A). These results and the fact that importin α1 also achieves the strongest signals in the mass spectrometric analyses (Fig. 6A; Table S2) encouraged us to map the domain in CHD4 that interacts with importin α1. It turns out that the CHD4 deletion mutant CHD4 Δ aa 2–149 still interacts, but CHD4 Δ aa 217–360 fails to interact, with importin α1. The region of aa 217–360 encompasses the nuclear import motif KRKR (Fig. 6D; Fig. S4A,B). Accordingly, the alanine mutant, CHD4 aa 350–354 Ala could still interact with importin α1, whereas CHD4 aa 304–307 Ala could not (Fig. 6D; Fig. S4A,B). Given that CHD4 Δ aa 217–360 and CHD4 aa 304–307 Ala were still able to move end-positioned nucleosomes on dsDNA, albeit with less activity than the wild-type protein (Fig. 3C; Fig. S4C), we propose that the missing or incorrect NLS is the reason for the inability of both proteins to interact with importin α1. This suggests that the NLS at aa 304–307 represents the interaction platform for importin α1. Furthermore, these results also argue against there being a NLS in the C-terminal part of the protein. The *in vitro* immunoprecipitations do not detect an importin α interaction using the CHD4 Δ aa 217–360 and CHD4 304–307 Ala (Fig. 6D).

Finally, we show that importin β1 does not interact with CHD4 or the CHD4 mutants in the *in vitro* pulldown assays (Fig. 6E; Fig. S4B), which confirms our *in vivo* data (Fig. 6A,B; Table S2). Taken together, we propose that the direct interaction of importin α1 with the monopartite NLS (aa 304–307) leads to nuclear import of human CHD4 (Figs 4, 6; Table S2, Fig. S4B), in addition to the piggyback mechanism discussed above (Fig. 5). Our data rather favor an importin β-independent transport route (Fig. 6A,B,E; Table S2, Fig. S4B).

## DISCUSSION

### The N-terminal amino acid region 304–307 contains a monopartite KRKR NLS

Nuclear proteins are usually only transferred to this compartment after complete translation (Hung and Link, 2011). Therefore, when searching for NLS motif(s), one must analyze the entire sequence of a protein (Table S1). For the human chromatin remodeler CHD4, we have successfully narrowed down the NLS to the monopartite N-terminal sequence KRKR (aa 304–307) using N- and C-terminal deletion mutants and alanine mutagenesis studies (Figs 2–4). The experimentally identified motif corresponds to the functionally relevant predictions of cNLS mapper (especially the monopartite ones) and NucPred, and is also highly conserved among homologous and paralogous CHD proteins (Table S1; Fig. S5). The functional activity of the NLS has been validated by attachment to a cytoplasmic resident 4×EGFP fusion protein and monitoring the import into the nucleus (Fig. 1F). In addition, a CHD4 mutant with the N-terminal amino acids 304–307 are mutated into alanine residues (CHD4 304–307 Ala) or where the N-terminal amino acid region aa 217–360 is deleted (CHD4 Δ aa 217–360), can no longer directly interact with importin α1 (Fig. 6D; Fig. S4B).

### CHD4 forms a complex with NuRD core proteins in the cytosol

Compared to what is seen for the wild-type protein, the nuclear localization of human CHD4 Δ aa 217–360 and 304–307 Ala is only reduced by ∼40–50% (Fig. 2C). However, our experiments do not suggest the existence of an additional, functional relevant NLSs in CHD4, given that mutation or deletion of the NLS in the full-length protein fully abrogates the interaction of CHD4 with importin α in *in vitro* immunoprecipitations (Fig. 6D). Thus, we propose that CHD4 can enter the nucleus through an additional mechanism also known as piggybacking (Hunter et al., 2014); CHD4 had already formed a complex with NuRD core subunits in the cytosol and thus these complex subunits could mediate nuclear import (Allen et al., 2013; Basta and Rauchman, 2015; Millard et al., 2016) (Fig. 5; Fig. S3). To our knowledge, we show for the first time that the association of cytosolic CHD4 with NuRD core subunits or possibly the assembly of a complete NuRD complex has already occurred in the cytoplasm of mammalian cells. Interestingly, Zhang and co-workers propose similar conclusions from work in *Drosophila* S2 Schneider cells (Zhang et al., 2016). By performing immunoprecipitation experiments with GFP-tagged p55 (a NuRD subunit) as a bait they present nuclear, but also cytosolic-resident ‘NuRD submodules’ with co-precipitated MTA-like, MBD-like and CHD4 (Zhang et al., 2016).

The passage of pre-assembled complexes through the nuclear pores is feasible and documented, with large RNA or DNA polymerase complexes, and large pre-ribosome complexes known to be exported through the nuclear pores (Alberts et al., 2002; Stawicki and Steffen, 2016). A structure in the center of the NPC seems to function like a tight-fitting membrane that opens just enough for the transport substrates to pass through, allowing an opening of the aperture from an average diameter of 9–10 nm to a diameter of ∼26 nm (Alberts et al., 2002; Stawicki and Steffen, 2016). This structure is formed by about ten different unfolded proteins (present in multiple copies), the so-called FG nucleoporins (Paci et al., 2020). Interestingly, it has been shown that the nuclear import of viral particles with diameters of 17–27 nm can be increased by increasing the number of NLSs. As larger molecules require more energy to pass through the nuclear pores, the increased number of NLSs can lead to increased binding of nuclear transport molecules (Paci et al., 2020). It is known that NuRD-associated nuclear subunits, such as HDAC1 or MTA proteins, possess intrinsic NLSs, which might therefore increase the import efficiency of CHD4–NuRD, supporting our suggestion that NuRD subunits might serve as a ‘second driving force’ besides the NLS in CHD4 (Brunmeir et al., 2009; Kumar and Wang, 2016; Paci et al., 2020).

The idea that NuRD subunits act as a ‘shuttle service’ in the CHD4 nuclear import process is further supported by the following findings. The NuRD core subunit RbAp48 (also known as RBBP4; with no own intrinsic NLS) has been shown to bind to the IBB domain of importin-α proteins in the presence of RanGTP, to induce the dissociation of importin β1 from importin-α proteins, thereby increasing the efficiency of the importin α and β-mediated nuclear import (Tsujii et al., 2015; Oka and Yoneda, 2018). In addition to RpAp48, the NuRD core subunit MBD2 has also been found to interact with importin-α proteins (Zhang and Li, 2010; Tsujii et al., 2015; Oka and Yoneda, 2018). Beyond that, interactome studies in T cells performed by Joshi and colleagues (Joshi et al., 2013) describe KPNA1, KPNA3 or KPNA6 (importin α5, α4 or α7) as binding partners of HDAC1 and HDAC2. Finally, CHD4 and the NuRD core subunits MTA2, HDAC2, p66 (GATA2B), RbAp48 and RbAp46 have been identified as some of the top 20 overlapping importin α7-binding partners by immunoprecipitation and pulldown assays (Hügel et al., 2014). These findings support the concept of CHD4 entering the nucleus by being piggybacked by NuRD core subunits.

### Different importin-α proteins might regulate the nuclear import of human CHD4

Interestingly, and in accordance with the findings of Hügel and colleagues (Hügel et al., 2014), importin α7 is also among the NuRD proteins co-precipitated with CHD4–GFP in our mass spectrometry analyses. We also detected importin α1, α5 and α6 alongside it. Importin α1, closely followed by importin α7, provides the strongest signals in the mass spectrometry analysis (Fig. 6A, Table S2 and Hoffmeister et al., 2017). Using western blotting, we also confirmed the co-precipitation of importin α1, α5 and α7 with CHD4–GFP (Fig. 6B).

The fact that more than one importin-α can associate with CHD4 has also been observed by Hügel and colleagues (Hügel et al., 2014), who proposed importin α2, along with importin α7, as a further interaction partner of CHD4. The slight differences in the detected importin proteins between our analyses and those of that publication (Hügel et al., 2014) might be explained by the use of different cell lines and experimental approaches. Given that several NuRD core subunits, such as RbAp48 or MBD2, can also interact with importin-α proteins (Zhang and Li, 2010; Tsujii et al., 2015; Oka and Yoneda, 2018), the fact that various importin-α proteins co-precipitate with CHD4–NuRD perfectly reflects the findings discussed above that an increase in NLSs in large molecules/complexes increases the number of binding transport proteins (Paci et al., 2020). Beyond that, it might also serve as a redundancy mechanism that could compensate for the loss of certain importin-α proteins with the remaining ones, as several importin-α proteins are characterized by a high degree of sequence similarity (Oka and Yoneda, 2018). Interestingly, proximity ligation mass spectrometry based on the BioID system in HEK 293 cells has shown that importin α1–BirA and importin α5–BirA fusion proteins share 478 of their interacting proteins, 179 of which are also detected with an importin α6–BirA fusion protein, arguing for certain functional redundancy of NTRs (Mackmull et al., 2017). The latter aspect is also confirmed by the data of Doyle et al. (2013), which show that knockdown of single importin-β or importin α7 or α8 do not significantly affect the nuclear accumulation of a dsRBD-PK-myc reporter-protein, since the respective other importin(s) probably compensate for the loss.

With regard to the functional significance of importin β1 in the transport of CHD4 to the nucleus, our *in vitro* data suggest enhancement or stabilization of CHD4–importin α1 complexes in the presence of importin β1 (Fig. 6C,D; Fig. S4B), as has been already shown for HIF2α (Depping et al., 2008). This finding is explained by the fact that the binding of importin β to the IBB domain of importin-α proteins eliminates the autoinhibitory effect of this domain, thus allowing easier access for NLS-containing proteins (Moroianu et al., 1996; Kobe, 1999; Kotera et al., 2005). However, neither our *in vitro* nor our *in vivo* studies show a direct or indirect association of CHD4–NuRD with importin β1 (Fig. 6; Table S2, Fig. S4B). Therefore, we assume an importin β1-independent, and thus exclusively importin α-dependent, nuclear import pathway for CHD4, as has been documented for other proteins (Kotera et al., 2005; Miyamoto et al., 2002; Oka and Yoneda, 2018). However, there are indications from other laboratories suggesting that the classical importin α and β pathway also takes place for CHD4–NuRD, as CHD4 has been identified as an interaction partner of importin β1 [also known as transportin (Trn)-1], using the SILAC-Tp method for different kind of HeLa extracts (Kimura et al., 2017). One explanation for the different findings could be the different experimental approaches or cell lines.

We propose that CHD4 is already present in the cytoplasm in conjunction with subunits of the NuRD nuclear complex. This, together with the direct interaction of, for example, importin α1 with the NLSs in the region 304–307, then ensures the import of CHD4–NuRD into the nucleus. Given that different physiological circumstances or cell and tissue types can influence the subunit composition of the NuRD complex (Denslow and Wade, 2007), it is conceivable that, on the one hand, the weighting between the piggyback mechanism and importin transport changes, but also that, in the case of the latter, the decision on the exclusive importin-α or the classical importin-α/β transport route fluctuates.

### Outlook

According to the Cosmic database (Forbes et al., 2017), we find cancer associated mutations in the KRKR NLS motif (aa 304–307) in human CHD4. Interestingly, only the arginine residue at aa position 305, which is definitely assigned functional relevance according to ConSurf and ConSeq (Fig. S5), seems to be affected by cancer associated mutations (mutations to cysteine or histidine residues). Interestingly, we can also see with CHD4 aa 304–307 Ala that the protein is less active than the wild-type protein. This leads to the assumption that the NLS has other functions besides transport. Something similar has been shown for IRF3, whose NLS also seems to play a role in the DNA binding of the protein (Zhu et al., 2015). One can easily imagine that the mislocalization of CHD4 or reduced CHD4 concentrations as a result of a mutated or defective NLS in combination with remodeling activity losses could result in chromatin structure changes, which in turn could lead to the development of diseases such as cancer (Hung and Link, 2011).

Furthermore, our data on the formation of NuRD core complex-like modules in the cytoplasm also leave room for speculation on CHD4 functions beyond the pure nuclear functions. SMARCAL1, another chromatin remodeling enzyme, has also been shown to be present in both the nucleus and the cytoplasm of HeLa cells in the G1 phase of the cell cycle, whereas it is significantly enriched in the nucleus in all other phases of the cell cycle (Haokip et al., 2016). Beyond that, FCS measurements in U2OS cells for nuclear, but as well cytosolic fractions of SNF2H–GFP or GFP–SNF2L have been published (Erdel et al., 2010). Finally, *Xenopus* CHD4 is localized to the nucleus during interphase, whereas it is cytoplasmic, but enriched on spindle MTs, during mitosis in *Xenopus* XL177 cells (Yokoyama et al., 2013).

Therefore, the in-depth study and characterization of NLSs in CHD4 and other remodelers will certainly help to better understand intracellular transport pathways and localization preferences in terms of functional significance. This in turn would also have a positive impact on understanding the dysfunctions of remodelers and how they can possibly be therapeutically counteracted.

## MATERIALS AND METHODS

### Reagents

Reagents used were: Gateway® BP Clonase® Enzyme Mix and Gateway® LR Clonase® Enzyme mix (Life Technologies); anti-HDAC1 antibody (Santa Cruz Biotechnology; sc-7872); anti-MTA2 antibody (Abcam; ab8106); anti-RbAp46 antibody (Abcam; ab3535); anti-TTF antibody (BD Biosciences; 611804); anti-tubulin antibody (Rockland; 200-301-880); anti-lamin A/C (Santa Cruz Biotechnology; sc-20681); 3H9 (anti-GFP) (Chromotek; 3H9); anti-importin α1 antibody (Everest Biotech; EB06233); anti-importin α5 antibody (Köhler et al., 1999); anti-importin α7 antibody (Köhler et al., 1999); anti-importin-β antibody (Santa Cruz Biotechnology; sc-1863); anti-Flag M2 antibody (F3165, Sigma); Tetra HIS antibody (Qiagen; 34670); GST (B-14) antibody (Santa Cruz Biotechnology; sc-138); goat anti-mouse-IgG peroxidase (HRP)-conjugated secondary antibody (cat. no. P0447, Dako, Hamburg, Germany); HRP-conjugated anti-rabbit-IgG (Jackson ImmunoResearch Laboratories; 111-035-144) and HRP-conjugated anti-mouse-IgG (Jackson ImmunoResearch Laboratories; 115-035-146); HRP-conjugated anti rat-IgG (Jackson ImmunoResearch Laboratories); HRP-conjugated anti-goat-IgG (Santa Cruz Biotechnology; sc-2020); GFP-Trap® A Beads and Binding control agarose beads (BAB-20) for preclearing (from Chromotek); anti FLAG® M2 Affinity Gel and FLAG® Peptide (Sigma-Aldrich); Amicon Ultra Centrifugal Filter Unit [MWCO 10 kDa] (Millipore); BSA (protease free, fatty acid free, essentially globulin free; Sigma-Aldrich); gelatin from cold water fish skin (Sigma-Aldrich); NuPAGE® Novex® 4–12% Bis-Tris Protein Gels, 1.0 mm, 10 well (Life Technologies); FCS (tetracycline free; Biochrom and Pan Biotech); FCS (Gibco); DMEM (1×)+GlutaMAX™-I (1 g*/*l glucose; Life Technologies); Gibco Sf-900™ II SFM (1×) serum-free medium (Life Technologies); doxycycline (Clontech Laboratories); hygromycin (Life Technologies); blasticidin (Life Technologies); FuGENE® HD transfection reagent (Promega); SuperSignalWest Dura Extended Duration Substrate and SuperSignalWest; Femto Trial Kit (Thermo Fisher Scientific); Triton X-100 (Sigma-Aldrich); Igepal CA-630 (Sigma-Aldrich); Tween-20 (Roth); 2-mercaptoethanol (Merck); Trypsin Gold, mass spectrometry grade (Promega); Benzonase ≥250 U*/*l (E1014 Sigma-Aldrich); ethidium bromide (Roth); NativePage® Novex® 4–16% Bis-Tris Gel (Thermo Fisher Scientific); ATP ultrapure (Jena Bioscience); DAPI (Sigma-Aldrich); Zero Blunt™ TOPO™ PCR Cloning Kit (Thermo Fisher Scientific); nitrocellulose membrane (Amersham Hybond-ECL, GE Healthcare); and PVDF membrane (Merck-Millipore).

### Plasmids

The pTetra-EGFP and pTetra-EGFP-SV40 vectors were a kind gift from Dr Christian Beetz (Friedrich Schiller University, Jena, Germany; Beetz et al., 2004). The NLSs covering aa 50–75, 111–134, 121–153, 301–311, 301–324 and 337–357 were ordered as ssDNA (oligonucleotide) forward and reverse sequences (Sigma-Aldrich), which were subsequently annealed to dsDNA oligonucleotide sequences, carrying 5′ EcoRI and 3′ BamHI ‘digest-overhangs’. The dsDNA oligonucleotide sequences were cloned into EcoRI and BamHI-digested pTetra-EGFP vector. The source(s) and cloning strategies for mammalian expression vectors (pEGFP-N1 GWc; pcDNA™5*/*FR*/*TO) encoding C-terminal GFP-tagged hCHD4, the insect cell expression vectors (pDFB6 C-Flag) encoding C-terminally Flag-tagged hCHD4, pOG44, and pPCRScript slo1-gla75 are described in detail by Hoffmeister and colleagues (Hoffmeister et al., 2017). The deletion mutant hCHD4 Δ aa 2–149 and Δ aa 217–360 were created with PCR according to Chiu and colleagues (Chiu et al., 2004) based on the respective pDONR™221 vector encoding the wild-type CHD4 protein (Hoffmeister et al., 2017). The alanine mutants in the context of hCHD4 (hCHD4 aa 304–307 Ala; aa 338–347 Ala; and aa 350–354 Ala) were generated by ordering dsDNA G-blocks from IDT (Coralville, Iowa 52241, USA) covering the respective cDNA part in hCHD4. The G-blocks were directly subcloned in pCR™Blunt II-TOPO® vector (Thermo Fisher Scientific), and finally subcloned via ApaI and PshAI digestion in pDONR™221 vector encoding the wild-type CHD4 protein (Hoffmeister et al., 2017). The mammalian (i) or insect (ii) expression vectors encoding C-terminal GFP (i)- or Flag (ii)-tagged hCHD4 alanine or deletion mutants (see above) were created by recombining the respective entry clones with pEGFP-N1 GWc or pDFB6 CFlag (Hoffmeister et al., 2017). The C-terminal deletion mutants hCHD4 aa 1–500 and aa 1–701 were created by amplifying the respective cDNA region from the pDONR™221 vector encoding the wild-type CHD4 protein (Hoffmeister et al., 2017). The PCR products were subsequently subcloned into pDONR™221 (Life Technologies) according to the BP recombination protocol from the Gateway® Technology manual (Life Technologies). The mammalian expression vectors encoding C-terminal GFP-tagged hCHD4 C-terminal deletion mutants were created by recombining the respective entry clones with pEGFP-N1 GWc (Hoffmeister et al., 2017). The plasmids encoding for recombinant proteins (GST, GST–α- or GST–β-importins, His-tagged importin-β and His-tagged nucleoplasmin), used in GST–importin pulldown assays, are as described by Depping and colleagues (Depping et al., 2008). All plasmids were sequence verified, and the oligonucleotide and G-block sequences are available upon request.

### Insect cell culture protein purification

*Spodoptera frugiperda* Sf21 cells were grown in Gibco Sf-900™ II SFM (1×) serum-free medium (Life Technologies) and as described by Hoffmeister and colleagues (Hoffmeister et al., 2017). The generation of virus coding for C-Flag hCHD4 and respective mutants was undertaken according to methods described by Fitzgerald et al. (2006) and Hoffmeister et al. (2017). Briefly, pDFB6 C-Flag with respective cDNA was transformed into chemically competent DH10Bac EM YFP (kindly provided by Dr Imre Berger, EMBL Grenoble, Grenoble Cedex 9, France; Fitzgerald et al., 2006). Positive transformants were screened via blue–white screening and used to isolate the bacmid DNA created by Tn7 transposition to transfect Sf21 cells using Fugene HD. The first virus generation (V_0_) was collected and subsequently used to produce the generation 1 virus (V_1_), which was used for large-scale infection of Sf21 cells. The infected cells were harvested by centrifugation (10 min, room temperature, 800 ***g***) at 48 h after the day of proliferation arrest. Approximately 2×10^8^ cells were lysed in 20 mM Tris-HCl pH 7.6, 500 mM KCl, 1.5 mM MgCl_2_, 0.5 mM EGTA, 10% glycerol and 0.1% Igepal CA-630, followed by three cycles of freezing and thawing in liquid nitrogen. The cell extract was sonicated using a Branson Sonifier 250 (Emerson Electric Co.) and centrifuged (20 min, 4°C, 18,000 ***g***). The supernatant containing the Flag-tagged proteins was incubated with 250 µl anti-FLAG® M2 Affinity Gel (gravity flow method). After washing the beads with lysis buffer and 20 mM TrisHCl pH 7.6, 333 mM KCl, 1.5 mM MgCl_2_, 0.5 mM EGTA, 10% glycerol and 0.1% Igepal CA-630, the protein was finally eluted in 20 mM Tris-HCl pH 7.6, 300 mM KCl, 1.5 mM MgCl_2_, 0.5 mM EGTA, 10% glycerol and 0.1% Igepal CA-630. 500 ng/µl FLAG® Peptide (5×150 µl). The protein concentration was determined via Bradford assay.

### Cell culture and transfection of mammalian cells

HeLa cells (ATCC® CCL-2™) were a kind gift from Dr Karsten Rippe (DKFZ, Bioquant Heidelberg, Germany) and were grown in DMEM with 10% FCS. The cells were transiently transfected with Fugene HD according to the manufacturer's instructions. Stably transfected Flp-In™ T-REx™ 293 cells (HEK 293-based cells; Life Technologies) that inducibly express C-GFP hCHD4 were generated as described by Hoffmeister and colleagues (Hoffmeister et al., 2017) and grown in DMEM with 10% tetracycline free FCS, 100 µg*/*ml hygromycin and 10 µg*/*ml blasticidin. Protein expression was induced by adding 1 ng*/*µl doxycycline for 24 h.

### Immunocytochemistry

At 48 h after transfection, cells were fixed in 4% PFA-PBS for 15–20 min. Afterwards, cells were washed three times in PBS and stained with DAPI (0.05 µg/ml in PBS) and washed again three times in PBS. Cells were finally mounted in 40–50% glycerin-PBS. Images were recorded using an Axiovert 200 M (Carl Zeiss AG) microscope and the Axiovision Rel. 4.7 software (Carl Zeiss AG). Data analysis in regard to intracellular localization was done manually by visualizing and counting the cells in the Axiovision Rel. 4.7 software according to Ding and coworkers (Ding et al., 2007; also see Results section). Cell-count data was visualized using R (https://www.r-project.org) with ggplot2 from the tidyverse collection of packages (https://www.tidyverse.org; Wickham et al., 2019) and ggpubr (https://rpkgs.datanovia.com/ggpubr/).

### Whole-cell extract preparation

At 48 h after transfection, cells were harvested by centrifugation (1600 ***g***) at 4°C and lysed in 20 mM Tris-HCl pH 7.6, 150 mM KCl, 1.5 mM MgCl_2_, 0.5 mM EGTA, 0.1–1% NP-40 (Igepal CA-630), 10% glycerol, 1 mM DTT, 1× complete, EDTA-free protease inhibitor cocktail (Roche; added according to the manufacturer's recommendations) for 10 min on ice. The cells were sonified for 5 min at level ‘high’ with an ultrasonic water bath system (Bioruptor; Diagenode) and centrifuged for 10 min at 4°C 16,000 ***g***. For western blot analysis, equal microgram amounts of whole-cell extract (WCE) were loaded onto denaturing SDS gels according to the Bradford measurement.

### Nuclear extract and cytoplasmic extract preparation

The preparation of cytoplasmic extract (CE) and nuclear extract (NE) extract was performed in according to Hoffmeister and colleagues (Hoffmeister et al., 2017). Briefly, Flp-In™ T-REx™ 293 stable expressing CHD4-GFP and GFP (i) or transiently transfected HeLa cells (ii) were lysed in 10 mM Tris-HCl pH 7.6, 3 mM MgCl_2_ and 10 mM NaCl with 0.5% Igepal CA-630 after 24 h induction with 1 ng*/*µl doxycycline (i) or 48 h after transfection (ii). The cell extract was centrifuged (10 min, 4°C, 500 ***g***) and the supernatant (CE) was collected. The nuclei pellet was washed twice with the above lysis buffer. The nuclei were finally lysed in 15 mM Tris-HCl pH 7.6, 60 mM KCl and 500 mM NaCl. After ultra-centrifugation (120,000 ***g***, 10 min, 4°C, rotor Beckman Coulter TLA100.4, Beckman Coulter Optima TL Ultracentrifuge 100,000 rpm) the supernatant (NE) was collected, and protein concentrations were estimated via Bradford assay. For comparative western blot analysis, equal microgram amounts of mutually corresponding CE and NE were loaded onto denaturing SDS gels according to the Bradford measurement.

### Immunopreciptiation of WCE, NE or CE

Co-immunoprecipitation (co-IP) experiments were performed according to Hoffmeister and colleagues (Hoffmeister et al., 2017). Briefly, co-IP experiments were performed with either 4–5 mg of WCE, CE or 1.5 mg NE using IP buffer [20 mM Tris-HCl pH 7.6, 150 mM KCl (for WCE and CE) or 150 mM NaCl (for NE), 1.5 mM MgCl_2_, 0.5 mM EGTA, 10% glycerol, 0.25% Igepal CA-630 and 1 mM DTT] in a total volume of 800 µl. The lysates were precleared in advance in IP buffer for 1 h at 4°C using 25 µl BAB-20 agarose beads. Afterwards, the precleared lysates were incubated with 25 µl GFP-Trap A with BAB-20 agarose beads for 3–4 h at 4°C on a rotating wheel. The GFP-Trap A and BAB-20 agarose beads were supplemented before with 1% gelatin and 200 ng/µl BSA in the respective IP buffer for 1 h at 4°C. If the lysates were further used for mass spectrometry analyses, GFP-Trap A beads and BAB-20 agarose beads were not blocked in advance. The beads were finally pelleted (2700 ***g***, 3 min, 4°C) and the supernatants were collected. The beads were subsequently washed once in IP buffer, once in IP buffer [500 mM KCl (for WCE and CE) or 500 mM NaCl (for NE)] and twice in IP buffer. The beads were finally resuspended in 2× Laemmli buffer. 40 µg (NE) or 70–80 µg (WCE and CE) of supernatant and input and 20–30% of one IP reaction were used for western blot analysis.

### Native protein gel electrophoresis and DLS

Oligomerization of CHD4 wild-type and mutants were assayed on 4–16% NativePAGE gels from Thermo Fisher Scientific according to Hoffmeister and colleagues (Hoffmeister et al., 2019). 1 μg protein was loaded in a total volume of 25 μl containing 6 μl NativePAGE 4× sample buffer and 2.4 μl NativePAGE 5% G-250 additive. 3 μl of Thermo Fisher Scientific NativeMARK unstained protein standard was used for better size estimation. Gels were run according to the manufacturer's instructions at room temperature, 150 V for 2 h using the dark blue cathode buffer protocol. After the run, gels were fixed in 40% methanol and 10% acetic acid and stained with Coomassie Brilliant Blue and subsequently with silver.

The size distribution of CHD4 and CHD4 mutants was determined by DLS (Panta by NanoTemper, Munich). Proteins were diluted in 1× PBS to a final concentration of 5 µM and analyzed in high-sensitivity glass capillaries (Nanotemper, Munich). Data analysis was performed with the Panta DLS software package (Nanotemper, Munich).

### Nucleosome assembly

The nucleosome assembly was performed via salt gradient dialysis. Briefly, an assembly reaction (40 µl) contained purified chicken histone octamers and DNA (both in the 2–20 µg range; prepared in-house; Maldonado et al., 2019) in ratios of 0.6–0.8:1 (histones:DNA) in high-salt buffer (10 mM Tris-HCl pH 7.6, 2 M NaCl, 1 mM EDTA, 1 mM 2-mercaptoethanol, 0.04–0.05% Igepal CA-630), supplemented with 200 ng*/*µl BSA. The template DNA for the assembly of 77-NPS-77 and 0-NPS-77 mononucleosomes was generated via PCR amplification using primers according to Hoffmeister and colleagues (Hoffmeister et al., 2017). The reaction was pipetted into a 1.5 ml Protein LoBind tube (Sarstedt), which was placed head-first in a foam-floater in 300 ml high-salt buffer. The tubes were manipulated in advance by introducing a hole of 6 mm diameter into the lid and by removing the bottom. Subsequently, a 1 cm^2^ piece of a buffer equilibrated dialysis membrane (MWCO 6–8 kDa) was mounted between lid and tube. After removing air bubbles between dialysis membrane and buffer, 3 l of low-salt buffer (10 mM Tris-HCl pH 7.6, 50 mM NaCl, 1 mM EDTA, 1 mM 2-mercaptoethanol and 0.05% Igepal CA-630) were pumped with a flow rate of 200 ml*/*h into the beaker containing the dialysis reaction. The success of an assembly reaction was checked by loading 50 ng of the nucleosomes (concentration determined via the applied DNA amount per reaction) on a PAA-gel, using non-assembled DNA as a control.

### Nucleosome remodeling reaction

A nucleosome remodeling assay was performed in 20 mM Tris-HCl pH 7.6, 150 mM KCl 1.5 mM MgCl_2_, 0.5 mM EGTA and 10% glycerol and 1 mM ATP at 30°C for 60 min in 10 µl volume containing 65–400 nM 0-NPS-77 mononucleosomes. The final concentration of recombinant nucleosome remodeling enzymes varied from 25 to 400 nM. The enzymatic reactions were stopped by adding 1000 ng competitor (plasmid) DNA for 5 min (at 30°C). The nucleosome movements were visualized by supplementing the reactions with glycerol (4–5%) and loading them on 6% PAA gels, which were subsequently stained with ethidium bromide.

### Mass spectrometry

One and a half IP reactions of a CHD4–GFP from nuclear extracts of stable transfected HEK 293 cells were loaded on a NuPAGE® Novex® 4–12% Bis-Tris Protein Gels, 1.0 mm, 10 wells. The gels were run in 1× MOPS buffer according to the manufacturer's protocol.

For mass spectrometric analysis (Hoffmeister et al., 2017), a gel lane was cut into 12 consecutive slices. The gel slices were then transferred into 2 ml microtubes (Eppendorf) and washed with 50 mM NH_4_HCO_3_, 50 mM NH_4_HCO_3_:acetonitrile (3:1) and 50 mM NH_4_HCO_3_:acetonitrile (1:1) while shaking gently in an orbital shaker (VXR basic Vibrax, IKA). Gel pieces were lyophilized after shrinking by 100% acetonitrile. To block cysteine residues, reduction with DTT was carried out for 30 min at 57°C followed by an alkylation step with iodoacetamide for 30 min at room temperature in the dark. Subsequently, gel slices were washed and lyophilized again as described above. Proteins were subjected to *in gel* tryptic digest overnight at 37°C with ∼2 µg trypsin per 100 µl gel volume (Trypsin Gold, mass spectrometry grade, Promega). Peptides were eluted twice with 100 mM NH_4_HCO_3_ followed by an additional extraction with 50 mM NH_4_HCO_3_ in 50% acetonitrile. Prior to liquid chromatography tandem mass spectrometry (LC-MS/MS) analysis, combined eluates were lyophilized and reconstituted in 20 µl of 1% formic acid. Separation of peptides by reversed-phase chromatography was carried out on an UltiMate 3000 RSLCnano System (Thermo Fisher Scientific, Dreieich), which was equipped with a C18 Acclaim Pepmap100 preconcentration column (100 µm i.d.×20 mm, Thermo Fisher Scientific) in front of an Acclaim Pepmap100 C18 nano column (75 µm i.d.×150 mm, Thermo Fisher Scientific). A linear gradient of 4% to 40% acetonitrile in 0.1% formic acid over 90 min was used to separate peptides at a flow rate of 300 nl/min. The LC-system was coupled on-line to a maXis plus UHR-QTOF System (Bruker Daltonics, Bremen) via a CaptiveSpray nanoflow electrospray source (Bruker Daltonics). Data-dependent acquisition of MS/MS spectra by CID fragmentation was performed at a resolution of minimum 60,000 for MS and MS/MS scans, respectively. The MS spectra rate of the precursor scan was 2 Hz processing a mass range between *m*/*z* 175 and *m*/*z* 2000. Via the Compass 1.7 acquisition and processing software (Bruker Daltonics) a dynamic method with a fixed cycle time of 3 s and an *m*/*z-*dependent collision energy adjustment between 34 and 55 eV was applied. Raw data processing was performed in Data Analysis 4.2 (Bruker Daltonics), and Protein Scape 3.1.3 (Bruker Daltonics) in connection with Mascot 2.5.1 (Matrix Science) facilitated database searching of the Swiss-Prot *Homo sapiens* database (release-2020_01, 220,420 entries). Search parameters were as follows: enzyme specificity trypsin with 1 missed cleavage allowed, precursor tolerance 0.02 Da, MS/MS tolerance 0.04 Da, carbamidomethylation or propionamide modification of cysteine, oxidation of methionine, deamidation of asparagine and glutamine were set as variable modifications. Mascot peptide ion-score cut-off was set 25. Search conditions were adjusted to provide a false discovery rate of less than 1%. Protein list compilation was undertaken using the Protein Extractor function of Protein Scape. Furthermore, we considered protein identification as confident, if the following criteria were met: at least 2 unique peptides found and a minimum protein score of 30. If necessary, fragment spectra were validated manually. EmPAI-values (exponentially modified protein abundance index), which can be used for an approximate relative quantitation of proteins in a mixture, were extracted from Mascot.

### GST–importin pulldown assay

The purification procedure for the recombinant proteins used for this assay are described by Depping and colleagues (Depping et al., 2008). Furthermore, GST–importin pull-down assays were carried out as described earlier (Depping et al., 2008). In brief, purified GST served consistently as a negative control. GST or GST–importins were allowed to bind to glutathione–Sepharose 4B (GE Healthcare). In a typical experiment 100 μl beads were pre-equilibrated in IP-buffer (20 mM HEPES pH 7.5, 100 mM KOAc, 0.5 mM EGTA, 5 mM MgOAc, 250 mM sucrose, 4°C), mixed with 15 μg GST fusion proteins and His-tagged importin-β, followed by an incubation step at 4°C overnight. After incubation, purified target CHD proteins (1.5 µg) were allowed to bind to the immobilized fusion proteins. In competition experiments, purified nucleoplasmin and importins were added in a 1:1 (molar) ratio. After several washing steps (with IP buffer), Sepharose beads were dissolved in 30 μl Laemmli buffer (Laemmli, 1970) and the bead fractions were subsequently analyzed by western blotting.

### SDS-PAGE and western blot analysis

Proteins were separated by SDS-PAGE (either 4–12% or gels with fixed percentage) and transferred by semi-dry blotting onto nitrocellulose membranes or PVDF membranes. The membranes were blocked in 5% nonfat dry milk powder in either TBS containing 0.1% Tween-20 (TBST) or PBS containing 0.1% Tween-20 (PBST) for at least 1 h at 4°C or room temperature. After blocking, the membranes were treated with the primary antibody (diluted in 5% nonfat dry milk powder in TBST or PBST) with cautious shaking overnight (i) or for 1 h (ii) at 4°C (i) or at room temperature (ii), followed by incubation with a (HRP)-conjugated secondary antibody (diluted in 5% nonfat dry milk powder in TBST or PBST) for 1 h at room temperature. Immunoreactive proteins were detected using ECL detection reagents (see also the Reagents section). Note that empty lanes in the blots have been spliced out for display purposes in the figures.

### NLS prediction and estimation of protein conservation

NLS predictions were made with NucPred (https://nucpred.bioinfo.se/nucpred/; Brameier et al., 2007), NLStradamus with a two-state static HMM (http://www.moseslab.csb.utoronto.ca/NLStradamus/; Nguyen Ba et al., 2009) and cNLS mapper with a cut-off score of 7.0 searching for bipartite NLS with long linkers over the entire sequence (http://nls-mapper.iab.keio.ac.jp/cgi-bin/NLS_Mapper_form.cgi; Kosugi et al., 2009). Sequence conservation of CHD4 was predicted using the ConSurf server (Ashkenazy et al., 2016) running in ConSeq mode without a given structure and default settings (Berezin et al., 2004), utilizing UNIREF90 and MAFFT (https://consurf.tau.ac.il/consurf_index.php). Full results of that particular run are available upon request. Additional multiple sequence alignments (MSA) were performed with a selected set of CHD4 homologs and CHD3 and CHD5 paralogues using MAFFT (Katoh and Standley, 2013) and visualized with ggmsa (http://yulab-smu.top/ggmsa/index.html; Zhou et al., 2022) in R (https://www.r-project.org).

## Supplementary Material

10.1242/joces.260724_sup1 Supplementary information
