# Supplementary material for: Characterization of the nuclear import of the human CHD4–NuRD complex
Source: J Cell Sci. 2023 Apr 6;136(7):jcs260724. doi: 10.1242/jcs.260724 (PMC10112965; doi:10.1242/jcs.260724)
Supplement: Supplementary information [file joces-136-260724-s1.pdf]

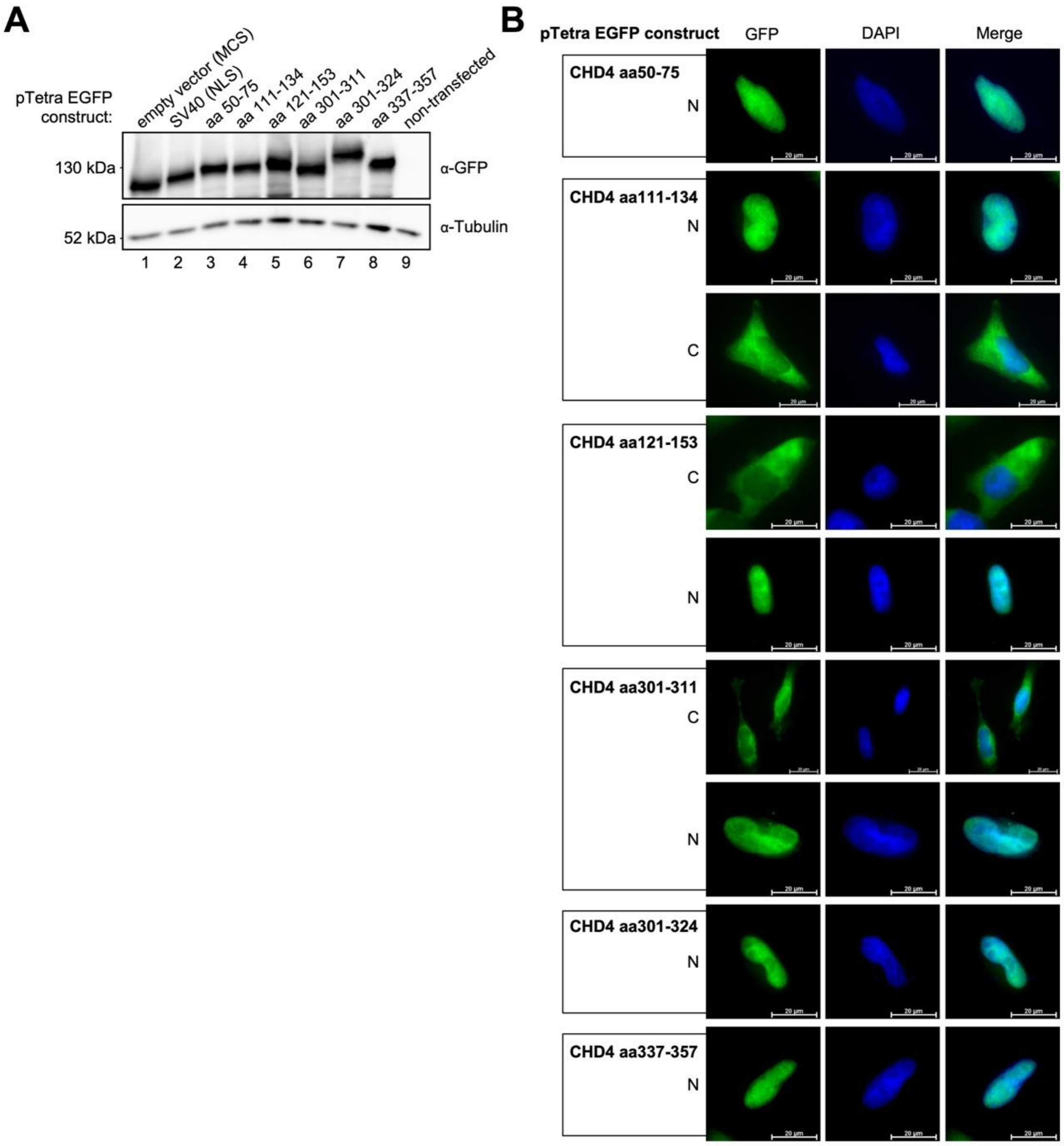

Fig. S1. Western blot analysis of HeLa cells expressing 4xEGFP with or without SV40 NLS or putative CHD4 NLS

(A) HeLa cells were transiently transfected with the empty pTetra-EGFP vector or pTetra-EGFP vectors encoding the 4xEGFP protein with SV40 NLS (see also Figure 1E) or aa 50-75; 111-134; 121-153; 301-311; 301-324; or 337-357 of human CHD4. Two days after transfection were harvested and lysed for whole cell extract preparation. Equal amounts according to Bradford were loaded for Western blot analysis with an anti GFP antibody. Tubulin served as a loading control. (B) HeLa cells were transiently transfected with the constructs in (A). Two days after transfection, the cells were fixed and stained with DAPI. In the panels we deposited representative pictures, reflecting the preferred localization of the respective CHD4 GFP fusion protein according to Figure 1E, F. Scale bars represent 20 µm.

Journal of Cell Science • Supplementary information

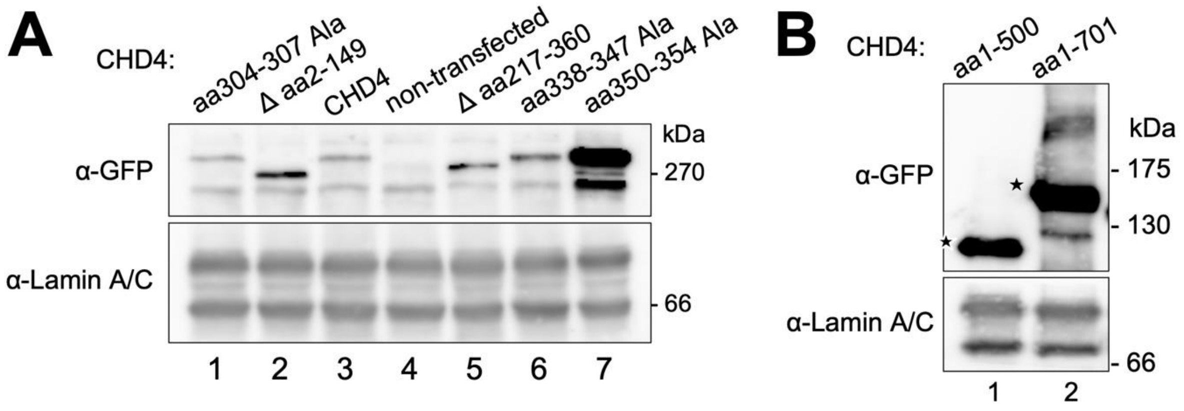

Fig. S2. Western blot analysis of HeLa cells expressing C-GFP hCHD4 wild-type, Δ aa 2-149, Δ aa 217-360, hCHD4 aa 304-307 Ala, aa 338-347 Ala, aa 350-354 Ala, and hCHD4 (1-500, 1-701)

(A) HeLa cells were transiently transfected with the constructs encoding C-terminal GFP tagged hCHD4 Δ aa 2-149, Δ aa 217-360, hCHD4 aa 304-307 Ala, aa 338-347 Ala, and aa 350-354 Ala. Two days after transfection, cells were harvested and lysed for whole cell extract preparation. Equal amounts according to Bradford were loaded for Western blot analysis with an anti GFP antibody. Lamin A/C served as a loading control. (B) HeLa cells were transiently transfected with C-GFP hCHD4 aa 1-500 and 1-701. Two days after transfection, cells were harvested and lysed for whole cell extract preparation. Equal amounts of whole cell extract according to Bradford were loaded for Western blot analysis with an anti GFP antibody (black asterisks thereby mark detected translation products with matching molecular weight). Lamin A/C served as a loading control.

Journal of Cell Science • Supplementary information

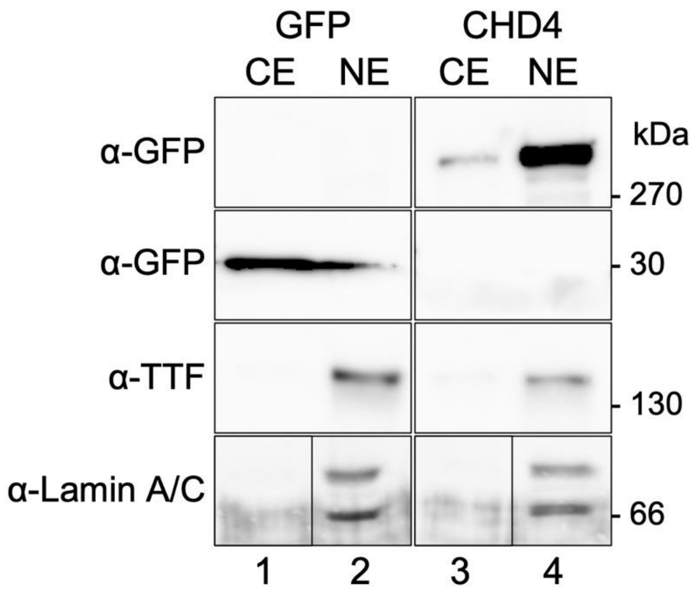

Fig. S3. Western blot analysis of cytoplasmic extracts from HEK 293 cells expressing C-GFP hCHD4 and GFP

Stable transfected HEK 293 cells expressing C-terminal GFP tagged CHD4 wild-type and GFP upon 24 hours of doxycycline induction were harvested and lysed for cytoplasmic extract preparation (see also Figure 5). Equal amounts of cytoplasmic- and (corresponding) nuclear extracts (arising from the preparation) of both cell lines were subjected to Western blot analysis with an anti GFP antibody. TTF and Lamin A/C stainings served as quality controls to ensure the depletion of nuclear proteins in the cytoplasmic extracts.

Journal of Cell Science • Supplementary information

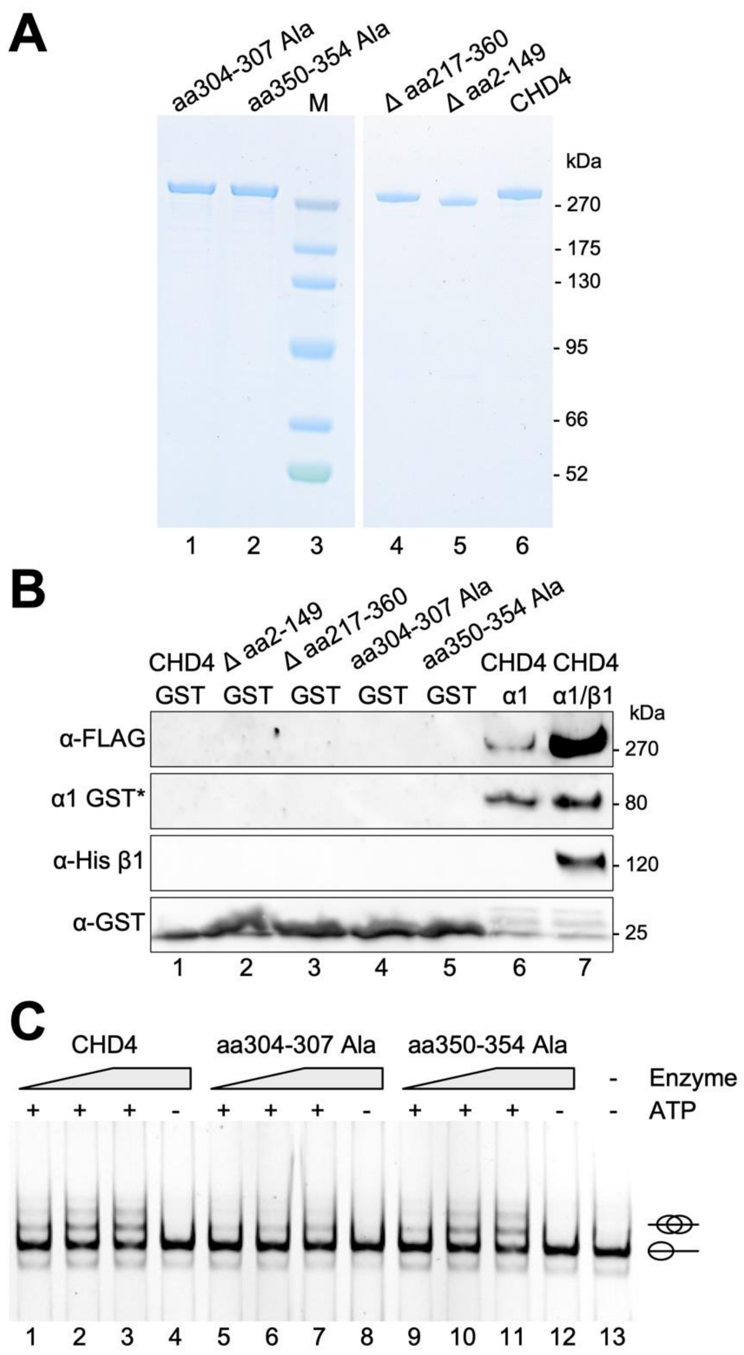

Fig. S4. Qualitative and functional analysis of purified human CHD4 wt and mutant proteins

(A) Human CHD4 C-Flag, CHD4 C-Flag Δ aa 2-149, CHD4 C-Flag Δ aa 217-360, CHD4 C-Flag aa 304-307 Ala, and CHD4 C-Flag 350-354 Ala were purified from Sf21 cells and purified via Flag M2-Beads. The proteins (600 ng each) were analyzed on a SDS page with subsequent Coomassie stain. (B) GST alone or GST-importin alpha 1 (α1) were immobilized in the absence or presence of His-tagged importin-β (β1) to Glutathione sepharose beads. Subsequently, purified wild-type CHD4-C-Flag, CHD4-C-Flag Δ aa 2-149, CHD4-C-Flag Δ aa 217-360, CHD4-C-Flag aa 304-307 Ala, or CHD4 C-Flag aa 350-354 Ala were allowed to

Journal of Cell Science • Supplementary information

bind to the immobilized fusion proteins. The bead fractions were resolved on SDS gels and analyzed by Western blot with an antibody directed against the Flag epitope (CHD4), an anti-His antibody (importin beta), or an anti GST antibody (GST protein itself). Note that there is a small amount of “co-purified” GST in all GST fusion protein fractions (lanes 6-7), which is detected by the GST antibody as well. However, the signal intensity for GST (lanes 1-5) is greatly enhanced in comparison to the GST by-product in all GST fusion protein fractions (lanes 6-7). Also note that the anti-Flag antibody also detects (non-specifically) the precipitated GST tagged importin alpha proteins (labeled with an asterisk). (C) 65 nM, 125 nM, and 250 nM of C-terminal Flag tagged, recombinant hCHD4 aa 304-307 Ala, hCHD4 aa 350-354 Ala, and hCHD4 wild-type were incubated with 80 nM 0-NPS-77 nucleosomes in the presence of 1 mM ATP for one hour at 30°C. After stopping the reactions, all samples were loaded on a 6% native gel, which was subsequently stained with ethidium bromide. Pictures for the purified nuclear import proteins used in the GST pulldown-assays were already shown by Depping and colleagues (Depping et al., 2008).

Journal of Cell Science • Supplementary information

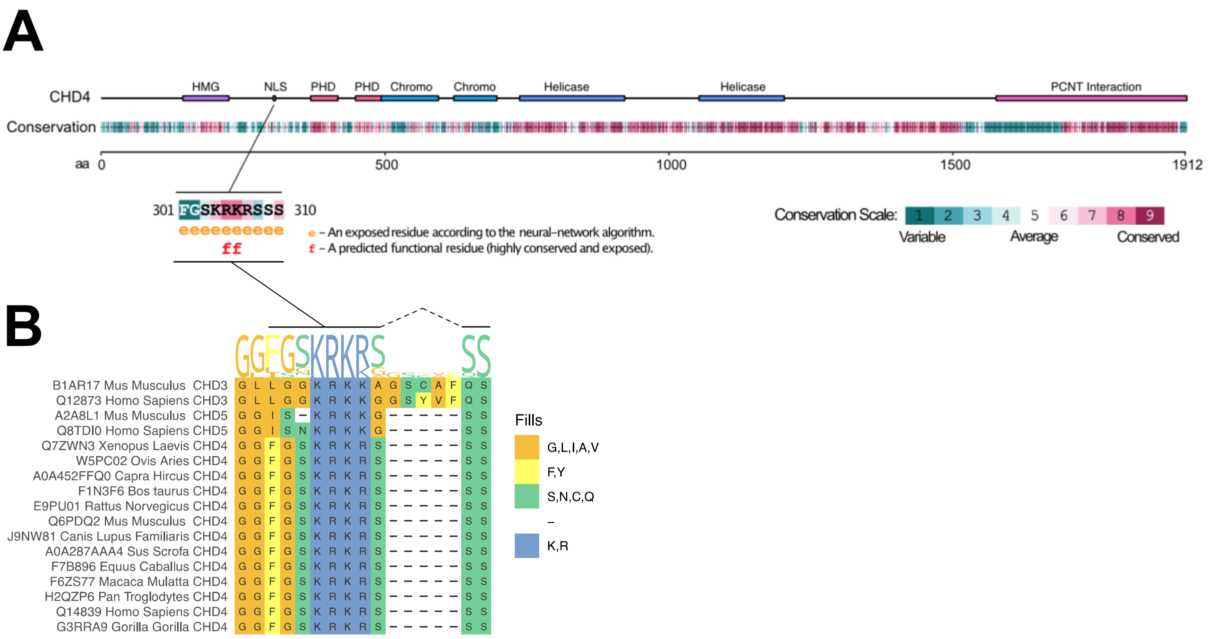

Fig. S5. Prediction of conserved regions of human CHD4 via ConSurf/ConSeq

(A) Conservation of residues in the complete aa sequence of human CHD4 (UniProt Q14839; 1912 aa) was predicted using the ConSurf-Server with default settings. Conservation is indicated by color scale from dark teal (variable; 1) over white (average; 5) to dark magenta (conserved; 9). Above the plot of the degree of conservation of hCHD4 is a plot of functional domains annotated by UniProt or Silva and colleagues (Silva et al., 2016). A 10-amino acid region including the NLS at aa 304-307 is shown enlarged in reference to the degree of conservation. The predicted exposure of the residue is indicated by a yellow "e" (exposed) and predicted functional residues are indicated by a red "f". (B) Multiple sequence alignment of selected CHD4 homologues and paralogues with a cumulative sequence logo on top. The region around the hCHD4 NLS at aa 304-307 are shown and amino acids are colored according to their side-chain chemistry. Uniprot accession numbers are given in front of the organism names.

Table S1. Detailed sequence- and score output of the three NLS prediction programs

(A) NLS sequences in human CHD4 predicted with NLStradamus using a posterior prediction cutoff of 0.6. (B) Monopartite and bipartite NLS sequences in human CHD4 predicted with cNLS mapper. Higher score indicates stronger NLS activities. (C) Influence of subsequences on nuclear localization predicted by NucPred. Colour scale indicates direction of influence and it is a gradient from blue (negative influence) over green (neutral) to red (positive influence).

Click here to download Table S1

Table S2. Mass spectrometry based list of all detected binding partners of hCHD4 C-GFP, which was precipitated from nuclear extracts of stable transfected HEK 293 cells

Stable transfected HEK 293 cells expressing C-terminal GFP tagged CHD4 wild-type upon 24 hours of doxycycline induction were harvested and lysed for nuclear extract preparation. Equal amounts of nuclear extracts were subjected to IP with GFP-Trap® A (= GFP)- and binding control agarose beads (BAB) (= Ctrl). Beads were finally dissolved in 2x Laemmli buffer and loaded on denaturing SDS gels, which were subjected to mass spectrometric analyses. A quantitative difference in protein amount levels is reflected by the emPAI-values (exponentially modified protein abundance index) (Ishihama et al., 2005). We considered protein identification as confident, if at least two unique peptides were found and the protein received a minimum protein score of 100. Data excerpted from this list can be found in Figure 6A.

Click here to download Table S2

Journal of Cell Science • Supplementary information
